# Supplementary material for: The hierarchy of sugar catabolization in Lactococcus cremoris
Source: Microbiol Spectr. 2023 Oct 27;11(6):e02248-23. doi: 10.1128/spectrum.02248-23 (PMC10715065; doi:10.1128/spectrum.02248-23)
Supplement: Supplemental Material — This document contains all supplemental information and figures including captions. [file spectrum.02248-23-s0001.pdf]

Supplemental Materials

The hierarchy of sugar catabolization in *Lactococcus cremoris*

Sieze Douwenga*[1,2], Berdien van Olst*[1,3,4], Sjef Boeren[1,4], Yanzhang Luo[5], Xin Lai[2], Bas Teusink[1,2], Jacques Vervoort[1,4,#], Michiel Kleerebezem[1,3], Herwig Bachmann[1,2,6]

*These authors contributed equally to this work

[1] TI Food and Nutrition, 6709 PA, Wageningen, the Netherlands

[2] Systems Biology Lab, Vrije Universiteit Amsterdam, 1081 HZ, Amsterdam, the Netherlands

[3] Host-Microbe Interactomics, Wageningen University & Research, 6700 HB Wageningen, The Netherlands

[4] Laboratory of Biochemistry, Wageningen University & Research, 6700 HB Wageningen, The Netherlands

[5] MAGNEtic resonance research FacilitY (MAGNEFY), Wageningen University & Research, 6708 WE, Wageningen, The Netherlands

[6] Microbiology Department, NIZO, 6718 ZB, Ede, The Netherlands

[#] Deceased

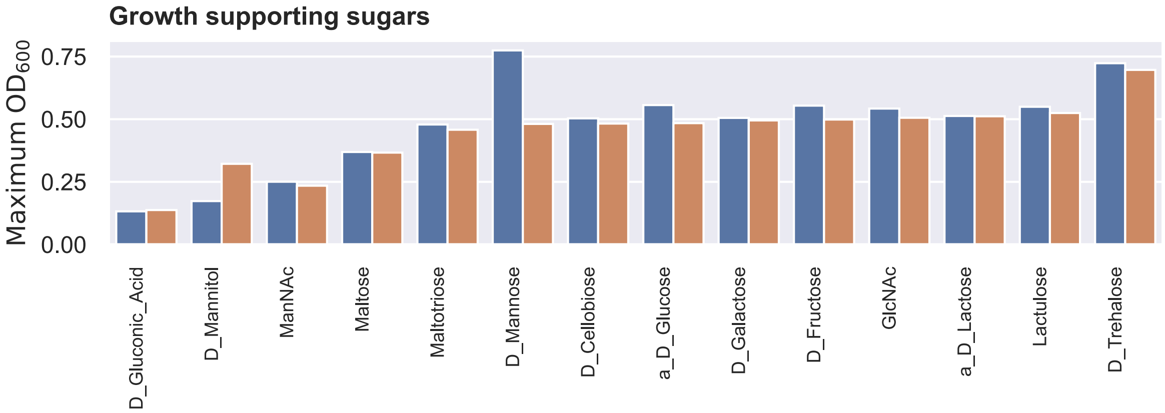

Figure S 1: Maximum OD600 reached through 30 hours of growth on the different sugars in a BiologTM plate. Sugars for which an OD600 > 0.2 was reached for both replicates (orange and blue) were considered to be growth supporting. D_GlcNAc=N-acetyl-glucosamine, ManNAc=N-acetyl-mannosamine. Growth on cellobiose was only observed after 24 hours for a subpopulation of cells (based on colony forming units on glucose and cellobiose plates). This is likely the result of a subpopulation of mutants capable of growing on cellobiose and not the entire L. cremoris NCDO712 population, similar to what was found for L. cremoris MG1363 (19). Hence, cellobiose was excluded for the remainder of the study. Additionally, maltotriose was excluded, as the BiologTM plate wells containing maltotriose were found to also contain some glucose (55-139 µM), meaning we could not discern maltotriose catabolic activity from glucose activity in these wells. The following carbon sources did not support growth: 1,2-Propanediol, 2-Aminoethanol, 2-Deoxy-Adenosine, Acetic acid, Acetoacetic acid, Adenosine, Adonitol, alfa-Hydroxy-Butyric acid, alfa-Hydroxy-Glutaric acid-gamma-Lactone, alfa-Keto-Butyric acid, alfa-Keto-Glutaric acid, alfa-Methyl-D-Galactoside, beta-Methyl-D-Glucoside, Bromo-Succinic acid, Citric acid, D,L-alfa-Glycerol-Phosphate, D,L-Malic acid, D-Alanine, D-Aspartic acid, D-Fructose-6-Phosphate, D-Galactonic acid-alfa-Lactone, D-Galacturonic acid, D-Glucosaminic acid, D-Glucose-1-Phosphate, D-Glucose-6-Phosphate, D-Glucuronic acid, D-Malic acid, D-Melibiose, D-Psicose, D-Ribose, D-Saccharic acid, D-Serine, D-Sorbitol, D-Threonine, D-Xylose, Dulcitol, Formic acid, Fumaric acid, Glucuronamide, Glycerol, Glycolic acid, Glycyl-L-Aspartic acid, Glycyl-L-Glutamic acid, Glycyl-L-Proline, Glyoxylic acid, Inosine, L-Alanine, L-Alanyl-Glycine, L-Arabinose, L-Asparagine, L-Aspartic acid, L-Fucose, L-Galactonic acid-gamma-Lactone, L-Glutamic acid, L-Glutamine, L-Lactic acid, L-Lyxose, L-Malic acid, L-Proline, L-Rhamnose, L-Serine, L-Threonine, m-Hydroxy-Phenyl-Acetic acid, m-Inositol, m-Tartaric acid, Methyl-Pyruvate, Mono-Methyl-Succinate, Mucic acid, Negative-Control, p-Hydroxy-Phenyl-Acetic acid, Phenylethyl-amine, Propionic acid, Pyruvic acid, Succinic acid, Sucrose, Thymidine, Tricarballylic acid, Tween-20, Tween-40, Tween-80, Tyramine, Uridine.

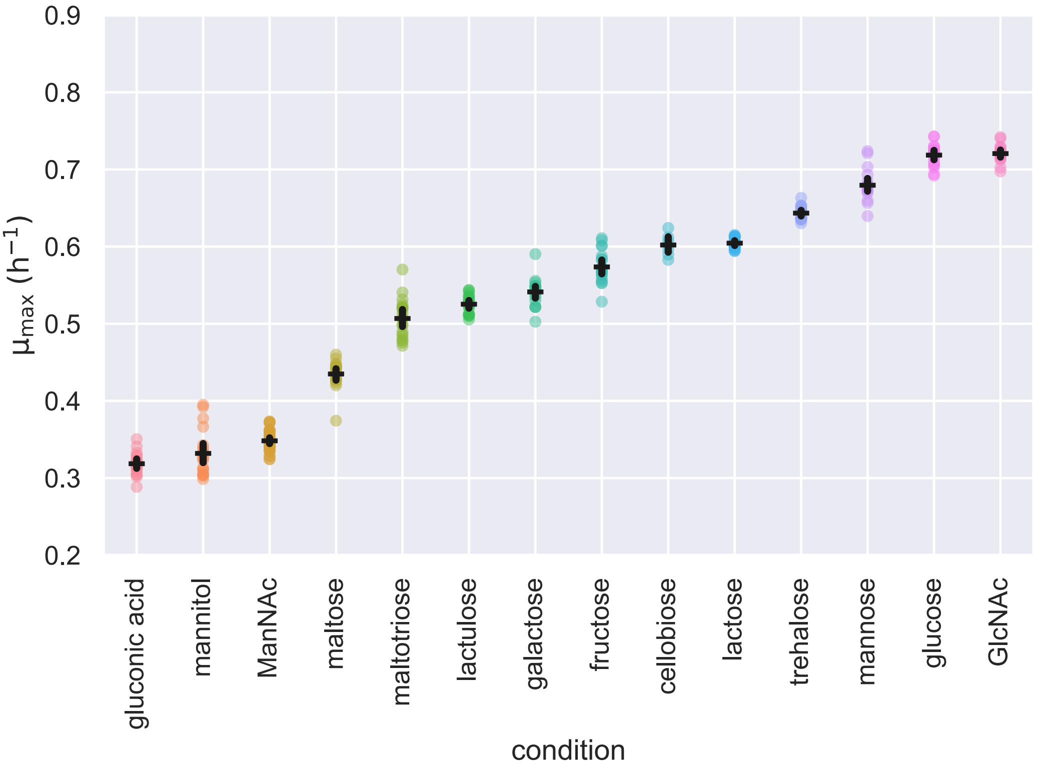

Figure S 2: Maximum growth rates (μ_{max}) of L. cremoris NCDO712 in CDMpc medium supplemented with 0.5% w/v of the growth supporting sugars identified in the Biolog™ plate. Mean maximal growths (black horizontal lines) are shown with 95% confidence interval (black vertical lines). Replicates are depicted by colored transparent dots (color is purely aesthetic). (n=21 for N-acetyl-mannosamine (ManNAc), n=20 for the other sugars). GlcNAc = N-acetyl-glucosamine.

Comparison to *L. cremoris* MG1363

For *L. cremoris* NCDO712, we determined the repression hierarchy by looking at catabolization rates in a Biolog™ plate after growth in CDMpc with various sugars. For *L. cremoris* MG1363, we performed an analysis on transcriptome-based repression levels of CcpA (a CCR related protein in *L. cremoris*) observed by Zomer et al., during growth on glucose in M17 (21). From their transcriptome comparison of a mid-exponential growing wild-type strain to a *ccpA* deletion mutant, we conclude that repression strength of sugar catabolic genes increased in the following order: mannitol & galactose (roughly equal repression), trehalose & maltose (roughly equal repression), and fructose & glucose (roughly equal repression) (21). We compared this repression strength hierarchy of MG1363 with its sugar quality hierarchy (Figure S 3). Similar to NCDO712, for MG1363, the CcpA-related repression hierarchy does not always follow the sugar quality hierarchy. Trehalose repression is comparable to that of maltose, whereas trehalose quality is higher (Figure S 3). Similarly, both fructose and glucose operons are not repressed, even though glucose is of higher quality than fructose.

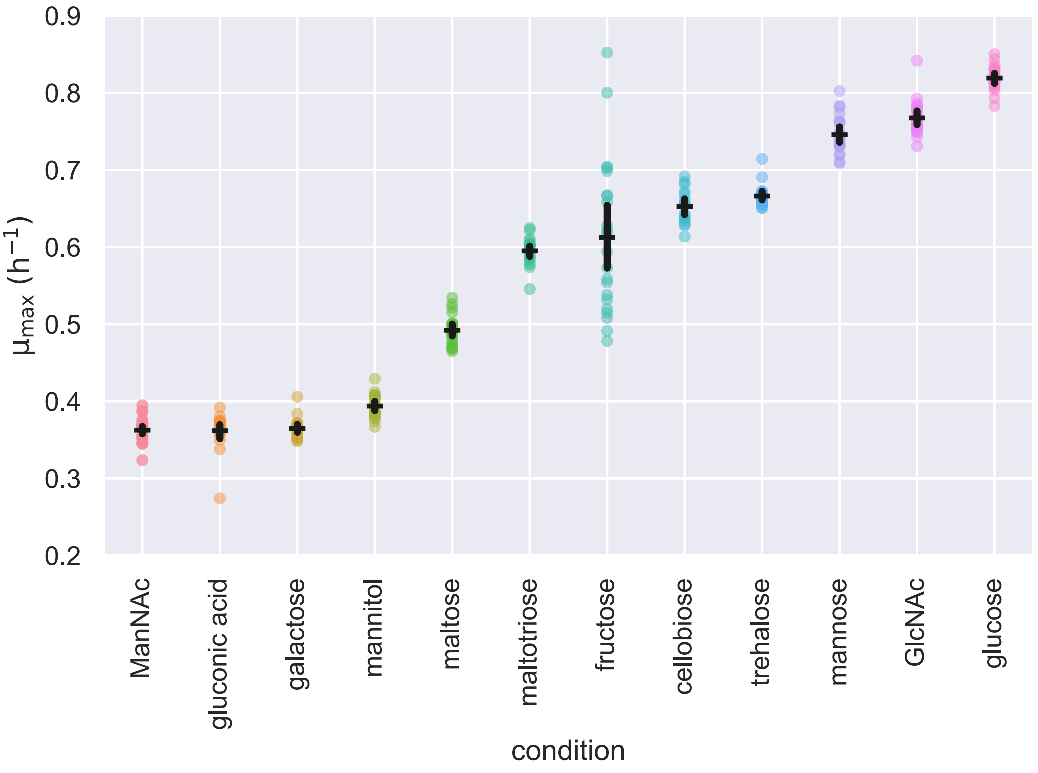

Figure S 3: Maximum growth rates (μ_{max}) of L. cremoris MG1363 in CDMpc medium supplemented with 0.5% w/v of the growth supporting sugars identified in the Biolog™ plate. Mean maximal growths (black horizontal lines) are shown with 95% confidence interval (black vertical lines). Replicates are depicted by colored transparent dots (color is purely aesthetic). (n=17 for cellobiose, n=22 for the other sugars). GlcNAc = N-acetyl-glucosamine; ManNAc = N-acetyl-mannoseamine.

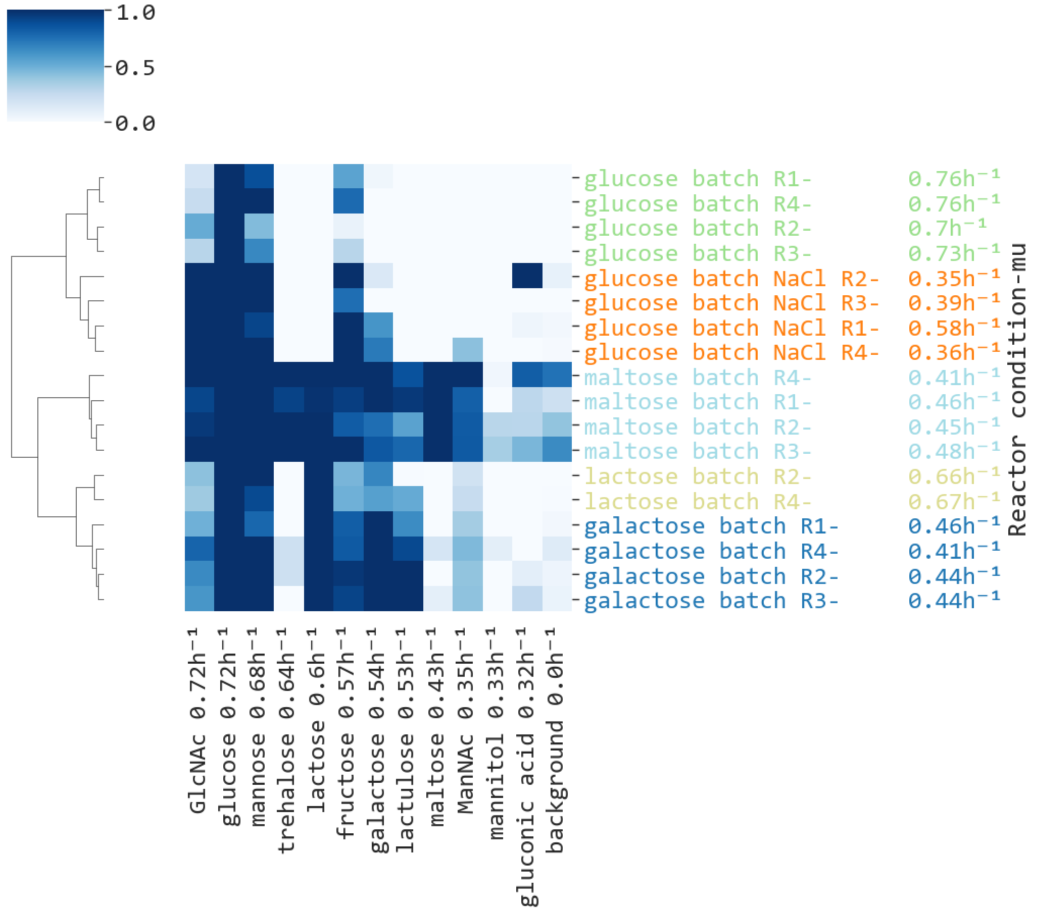

Figure S 4: Heatmap of normalized specific catabolization rates (color legend top left) of different growth supporting sugars in the Biolog™ plates (bottom, includes corresponding maximum achievable growth rate on each sugar as measured separately in microtiter plates, (Figure S2)) in the presence of chloramphenicol and erythromycin when growing under various conditions at different growth rates (right, text colors match growth conditions). Each row is the result of one Biolog™ plate. R1, R2, R3, and R4 indicate biological replicates (taken from different reactors), in the case of lactose Biolog™ plate samples could only be successfully taken from R2 and R4. Ward clustering based on the Euclidian distance between the different Biolog™ plates is shown on the left. GlcNAc=N-acetyl-glucosamine, ManNAc=N-acetyl-mannosamine.

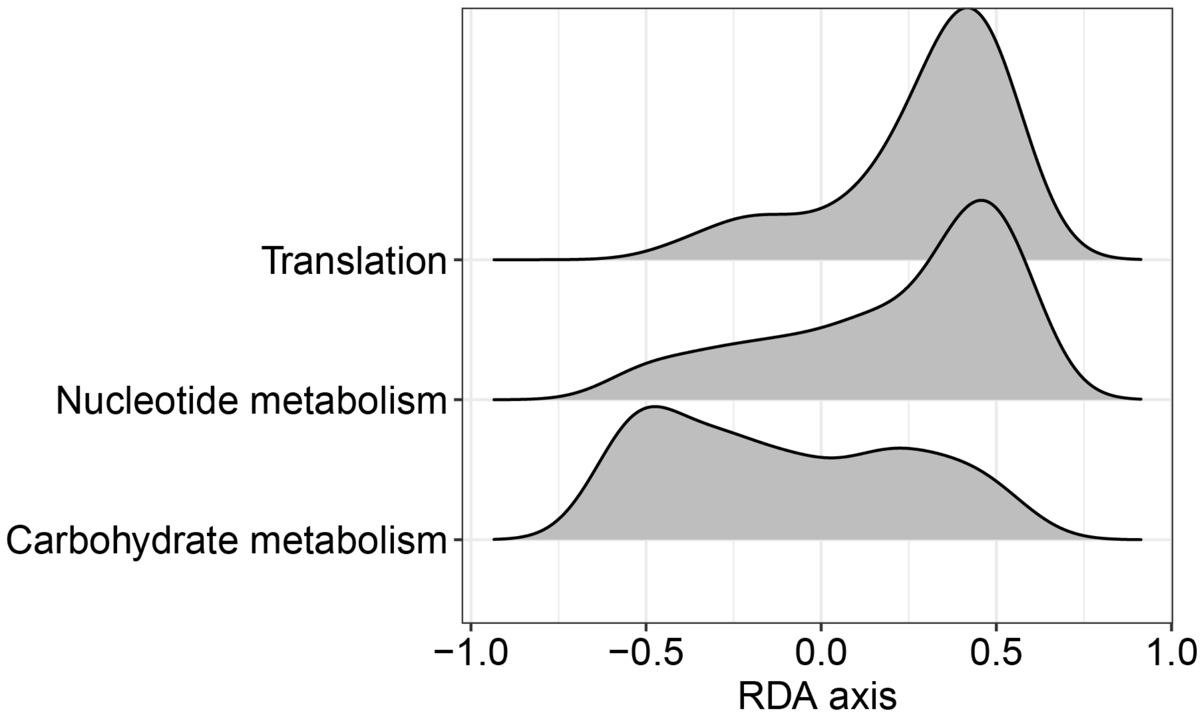

Figure S 5: Distributions of Brite hierarchy categories (y-axis) that were identified as enriched by gene set enrichment analysis on the RDA component associated with growth (x-axis). Positive and negative RDA-scores correlate with higher and lower growth rates, respectively.
